# TM6SF2 rs58542926 is related to hepatic steatosis, fibrosis and serum lipids both in adults and children: A meta-analysis

**DOI:** 10.3389/fendo.2022.1026901

**Published:** 2022-10-24

**Authors:** Xue-Ying Li, Zheng Liu, Li Li, Hai-Jun Wang, Hui Wang

**Affiliations:** ^1^Department of Maternal and Child Health, School of Public Health, Peking University, Beijing, China; ^2^Department of Endocrinology and Metabolism, Ningbo First Hospital, Ningbo, China

**Keywords:** *TM6SF2* rs58542926, NAFLD, alanine aminotransferase, serum lipid levels, meta-analysis

## Abstract

**Background and aims:**

Findings about the associations between transmembrane 6 superfamily member 2 (*TM6SF2*) rs58542926 and nonalcoholic fatty liver disease have not been consistently replicated, particularly in steatosis and fibrosis. The present study aimed to investigate the associations between the rs58542926T allele and the spectrum of NAFLD and its related metabolic phenotypes.

**Methods:**

Systematic literature research was performed to analyse the associations between rs58542926 and the spectrum of NAFLD and its related metabolic phenotypes. A random effects meta-analysis with a dominant genetic model was applied.

**Results:**

Data from 123,800 individuals across 44 studies were included in the current meta-analysis.rs58542926 T allele was associated with an increased risk of NAFLD in both adults (OR=1.62; 95% CI: 1.40, 1.86) and children (OR=2.87; 95% CI: 1.85, 4.46). Children had a stronger association with NAFLD (*P*=0.01). rs58542926 T allele was also positively associated with steatosis progression (mean difference=0.22; 95% CI: 0.05, 0.39) and fibrosis stage (OR=1.50; 95% CI: 1.20, 1.88) in adults. The *TM6SF2* rs58542926 T allele was positively associated with ALT in both adults and children (both *P*<0.01) and only with higher AST in adults (*P*<0.01). The rs58542926 T allele was negatively associated with serum total cholesterol (TC), low-density lipoprotein (LDL), and triglycerides (TGs) in both adults and children (all *P*<0.01).The serum level of TG was much lower in adults than in children (*P*<0.01).

**Conclusion:**

*TM6SF2* rs58542926 is involved in the entire spectrum of NAFLD and its related metabolic phenotype, and differences in serum lipid levels were observed between adults and children.

**Systematic review registration:**

https://www.crd.york.ac.uk/PROSPERO/, identifier CRD42021288163.

## Introduction

Nonalcoholic fatty liver disease (NAFLD) is a complex disease that is closely related to genetic susceptibility and lifestyles. NAFLD has become the most common chronic liver disease worldwide in both adults and children (1, 2). The global prevalence of NAFLD is currently estimated to be 32.4% in adults and 7.4% in children (1, 3). NAFLD consists of a broad spectrum of liver diseases, including nonalcoholic fatty liver (NAFL), nonalcoholic steatohepatitis (NASH), liver cirrhosis and even hepatocellular carcinoma (HCC) (4). NAFLD is considered a risk factor for cardiovascular diseases and type 2 diabetes due to dyslipidemia and hyperglycemia (5).

In recent years, molecular epidemiological studies have suggested that gene variation plays an important role in the occurrence and development of NAFLD. A prospective twin study showed that the heritability of hepatic steatosis (based on MRI-PDFF) was 0.52 and the heritability of hepatic fibrosis (based on liver stiffness) was 0.5 (6). Identification and understanding of its related genetic variants are important for the treatment of hepatic steatosis and its advanced stages. With the implementation of a genome-wide association study (GWAS) on liver fat, more than twenty single nucleotide polymorphisms (SNPs) have been involved in the pathogenesis of NAFLD, for instance, rs738409 (C>G, pI148 M) of patatin-like phospholipase-domain-containing 3 (*PNPLA3*) and rs58542926 (C>T, pE167K) of transmembrane 6 superfamily member 2 (*TM6SF2*) (7).

SNP rs58542926 triggers hepatic fat accumulation by reducing very low-density-lipoprotein (VLDL)-mediated lipid secretion and increases the risk of lipid accumulation in hepatocytes and decreases the circulating lipids in serum (8). However, the relationships between the *TM6SF2* rs58542926 variant and predisposition to all spectra of NAFLD remain controversial in the current literature (9). Liu et al. (10) showed that the *TM6SF2* rs58542926 variant could affect the progression of fibrosis in European Caucasian NAFLD participants, but Wong et al. (11) showed that the *TM6SF2* rs58542926 variant did not cause liver fibrosis or cirrhosis in Chinese NAFLD subjects.

In addition, serum concentrations of alanine aminotransferase (ALT) and aspartate aminotransferase (AST) have been classically regarded as markers of liver function damage with NAFLD progression (12). A recent exome-wide association study of liver fat content showed that the *TM6SF2* rs58542926 variant was associated with ALT in the Dallas Biobank and the Copenhagen Study, but the relationship was not statistically significant in AST (13). However, these results were not confirmed in genome-wide association studies (GWASs) (14). However, some GWAS have suggested that the *TM6SF2* rs58542926 variant is closely associated with serum lipid levels (15–17). However, the results of these studies were conflicting and nonreplicated. In addition, whether those associations were consistent between adults and children is unknown.

Therefore, the present study aims to summarize all eligible results to clarify whether the *TM6SF2* rs58542926 variant influences the development of NAFLD and related metabolic phenotypes in both adults and children and to compare the differences in the effect values of the variant between adults and children.

## Methods

### Data sources and study selection

This meta-analysis followed the HuGENet and MOOSE reporting guidelines.

A comprehensive search for literature was conducted in the PubMed, EMBASE, Web of Science, The Cochrane Library and CNKI databases. The specific search strategy was “rs58542926 or E167K or Glu167Lys”. The search was completed on October 29, 2021. After removing duplicate literature, titles and abstracts were independently screened for eligibility by 2 authors, with inclusion/exclusion criteria applied to potentially eligible full texts.

### Inclusion and exclusion criteria of the literature

The study protocol, including the search strategy and inclusion and exclusion criteria, was registered on PROSPERO Database of Systematic Reviews (CRD42021288163). Inclusion criteria: (1) genetic association studies on the *TM6SF2* rs58542926 variant and NAFLD in human beings; (2) provide genotype/allele frequency of rs58542926 polymorphisms in the study population; (3) full text available in English or Chinese. Exclusion criteria: (1) case reports, reviews, meta-analyses, comments, and repeated published literature; (2) lack of detailed genotyping data or relevant outcomes; (3) *in vitro* and animal studies; (4) preprint and abstract publications; (5) studies conducted in patients with infectious liver disease (HBV or HCV infection).

### Study selection and data extraction

**Figure 1:**
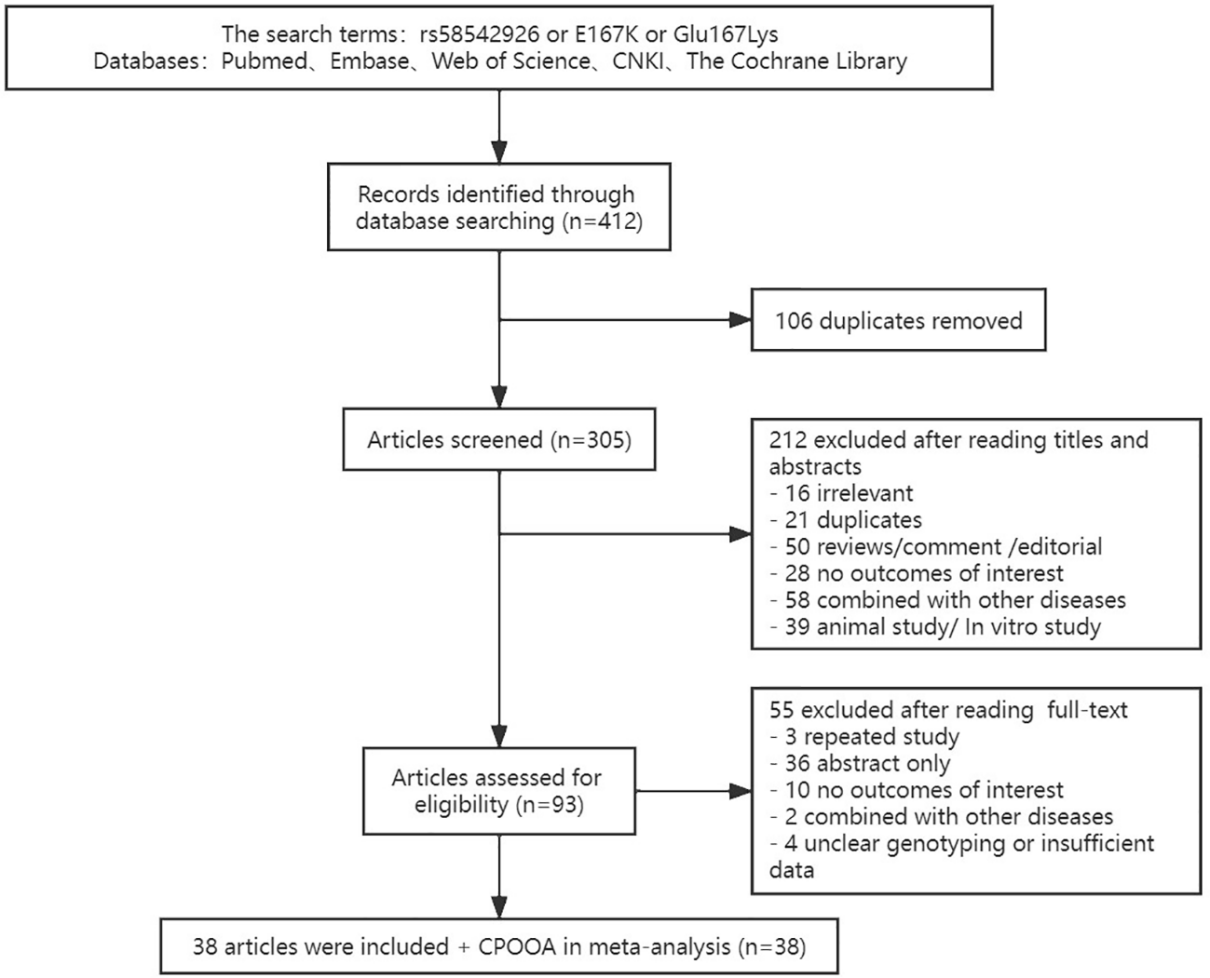
Flowchart of study selection.

Literature was initially selected based on title and abstract, and we reviewed the full texts to select qualified articles based on eligibility criteria. Study selection was performed by 2 independent reviewers. Any disagreement between the two reviewers was discussed with and resolved by a third investigator. Database searches identified 305 articles, of which 38 articles (10, 11, 13, 14, 18–50) met the inclusion criteria for pooled meta-analyses. In addition, we added our own sample results in the children part, which was named the Comprehensive Prevention Project for Overweight and Obese Adolescents (CPOOA) (51, 52) (**Figure 1**).

Information extraction was performed by 2 independent reviewers. , author’s name, publication year, country and ethnicity of study subjects, total numbers of participants, adults/children, NAFLD-related metabolic phenotypes [including serum concentrations of total cholesterol (TC), TG, low-density lipoprotein (LDL), high-density lipoprotein (HDL), ALT and AST], steatosis and fibrosis were extracted from the selected studies. In addition, the number of different genotypes of *TM6SF2* rs58542926 carriers and the results of the Hardy-Weinberg equilibrium (HWE) test were recorded.

### Statistical analyses

The Review Manager Version 5.4 was used to conduct statistical analyses and forest plots. Due to the low frequency of TT homozygous in *TM6SF2* rs58542926, genetic association analyses were performed with the dominant model.

NAFLD, hepatic steatosis and fibrosis stages were evaluated as dichotomous variables (as Yes/No), and the effect size was calculated as an odds ratio (OR) between groups. In addition, the progression of steatosis and fibrosis (the degree of hepatic steatosis, lobular inflammation and balloon degeneration) were treated as continuous variables. Serum lipid levels (TC, TG, LDL, HDL), ALT and AST were evaluated as continuous variables individually. For continuous variables, effect sizes were calculated as the mean differences (MD). All values of medians and the interquartile range or total range were converted to the mean and standard deviation.

Both the fixed- and random-effects models were explored for all outcome variables, and the random model was selected to represent final results, since it would make the results tend to be conservative with the test of Dersimonian Laird. The results were reported with effect sizes and 95% confidence intervals (CIs). *P* ≤0.05 was considered statistically significant. The fixation index was calculated to compare the differences in *TM6SF2* rs58542926 among different ethnicities and no differences was found (53). Considering the possible heterogeneity between adults and children, a subgroup analysis between children and adults was conducted. An additional subgroup analysis was performed for fibrosis stages (with fibrosis vs without fibrosis; fibrosis stage 0-1 vs 2-4; fibrosis stage 0-2 vs 3-4), and those analyses were only performed within individuals with NAFLD. Heterogeneity between groups was described using the Q statistic, tau^2^, and *I*^2^. In terms of forest plots, ORs were pooled using the method of inverse variance.

Two reviewers independently assessed the risk of bias of each study by applying the evaluation tool established by the Agency for Healthcare Research and Quality (https://effectivehealthcare.ahrq.gov/products/collections/cer-methods-guide) (54). All studies were assessed based on their design, either case−control or cross-sectional studies (**Figure S1**). Finally, the risk of bias was divided into three levels: low, high and unclear. The stabilities of pooled results were determined by omitting one study each time and pooling the results of the remaining studies. Funnel plots were constructed to test publication biases.

## Results

### Characteristics of selected studies

In total, 44 original studies (36 in adults and 8 in children) and 123,800 individuals (5432 children) were included in the current meta-analysis. Most of the studies were conducted in Europe (21 studies), 13 in Asia, 3 in North America and 2 in South America. The main characteristics of the studies are shown in **Table S1**. The summary of the results of the meta-analysis and the disparities between adults and children with NAFLD as well as its related metabolic phenotypes are presented in **Table 1**.

**Table 1:** Summary of results from meta-analysis for adults and children.

Outcome	Analysis	Subpopulation	Number of studies	Heterogeneity		Effect summary		Adults vs Children*
				I^2^	*P_Q_*	Estimate	*P_Z_*	*P*
NAFLD diagnosis	Control *vs.* NAFLD	Adult	14	0.37	0.07	1.62 (1.40, 1.86) ^†^	<0.01	
		Child	6	0.59	0.03	2.87 (1.85, 4.46) ^†^	<0.01	0.01
Steatosis	Severe (stage S0-S1 *vs.* stage S2-S3)	Adult	4	0.34	0.21	1.52 (1.14, 2.02) ^†^	<0.01	N/A
	Steatosis progression	Adult	55	0.8	<0.01	0.22 (0.05, 0.39) ^‡^	0.01	N/A
Fibrosis	Occurs (stage F0 *vs.* stage F1-F4)	Adult	2	0	0.95	1.07 (0.63, 1.82) ^†^	0.80	N/A
	Severe (stage F0-F1 *vs.* stage F2-F4)	Adult	5	0.58	0.05	1.46 (1.07, 1.99) ^†^	0.02	N/A
	Advanced (stage F0-F2 *vs.* stage F3-F4)	Adult	3	0	0.77	2.04 (1.36, 3.05) ^†^	<0.01	N/A
	Fibrosis progression	Adult	4	0.8	0.002	0.32 (0.03, 0.61) ^‡^	0.03	N/A
Aminotransferase	ALT	Adult	15	0.90	<0.01	3.34 (1.67, 5.00) ^‡^	<0.01	
		Child	4	0.13	0.33	3.93 (1.71, 6.16) ^‡^	<0.01	0.68
	AST	Adult	13	0.86	<0.001	1.91 (0.59, 3.23) ^‡^	<0.01	
		Child	2	0.44	0.18	2.90 (-1.58, 7.37) ^‡^	0.2	0.68
Serum lipids	TC	Adult	14	0.42	0.03	-10.01 (-12.46, -7.56) ^‡^	<0.01	
		Child	5	0.38	0.17	-10.85 (-14.64, -7.05) ^‡^	<0.01	0.72
	TG	Adult	16	0.76	<0.01	-14.36 (-18.71, -10.02) ^‡^	<0.01	
		Child	5	0	0.90	-6.55 (-10.00, -3.11) ^‡^	<0.01	<0.01
	LDL	Adult	13	0	0.54	-2.04 (-2.63, -1.44) ^‡^	<0.01	
		Child	4	0	0.41	-8.77 (-11.25, -6.30) ^‡^	<0.01	<0.01
	HDL	Adult	15	0.60	<0.001	0.66 (0.00, 1.32) ^‡^	0.05	
		Child	4	0.19	0.29	-0.81 (-2.16, 0.53) ^‡^	0.24	0.05

^*^: Differences between adults and children were detected with Chi square test.

† OR, 95% CI; ^‡^ Mean difference, 95% CI.

Meta-analyses were performed using random effects with subgroup analysis for adult and child populations. The results using a dominant model of inheritance (CC vs. CT + TT) are shown for all outcomes. PZ <0.05, significant. Meta-analyses were performed using random effects with the DerSimonian−Laird method for estimation of tau2; NAFLD, nonalcoholic fatty liver disease; ALT, alanine aminotransferase; AST, aspartate aminotransferase; TC, total cholesterol; TG, triglyceride; LDL, low-density lipoprotein; HDL, high-density lipoprotein; OR, odds ratio; CI, confidence interval.12; NA, Not Available.

### The rs58542926 T allele increased the risk of NAFLD in both adults and children

**Figure 2:**
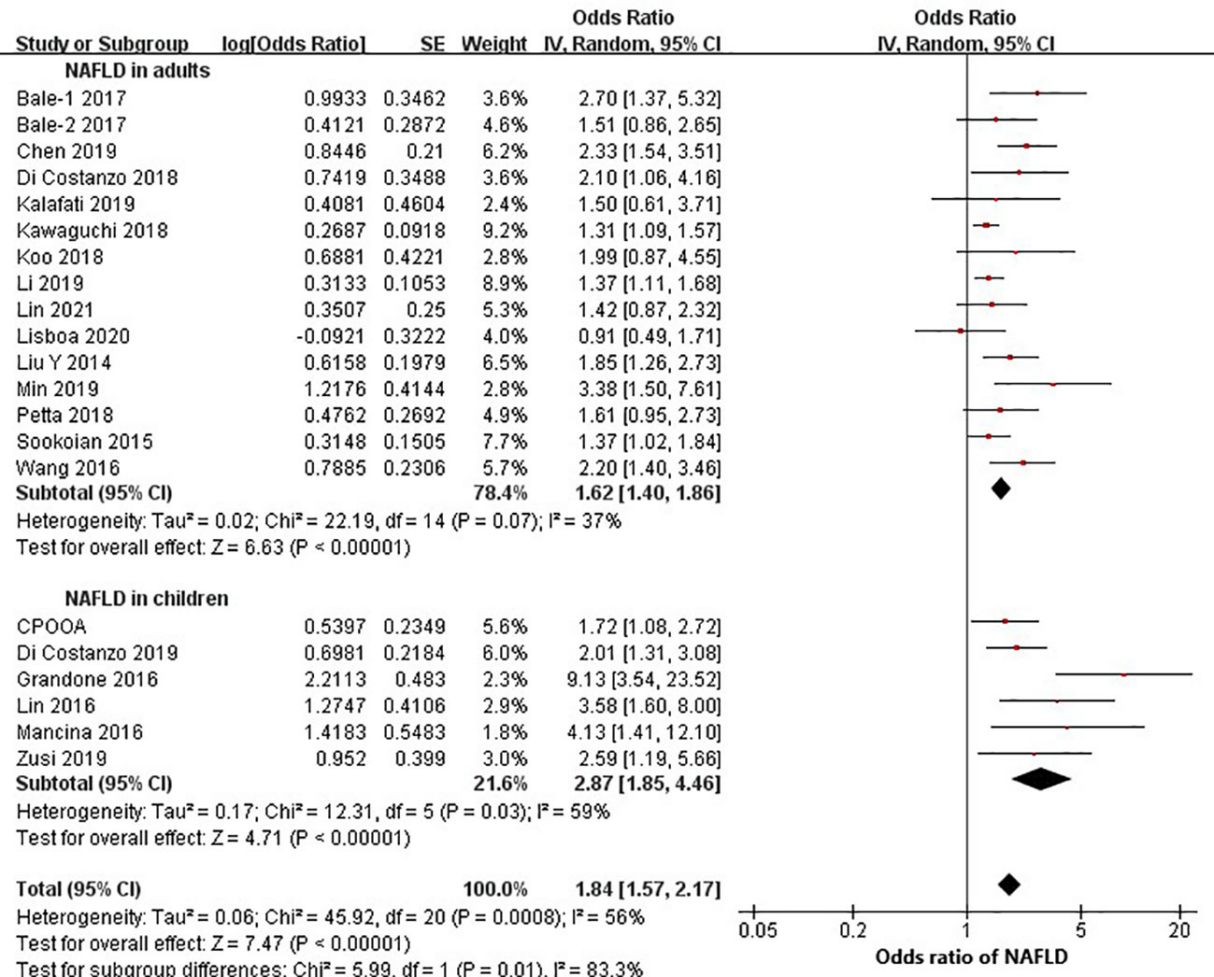
rs58542926 T allele was associated with a higher odds ratio of NAFLD. Data from 19,445 individuals (15,901 adults, 3544 children) with CT, MRI or FibroScan. rs58542926 T allele was positively associated with NAFLD in both adults and children (using a dominant model of inheritance). Meta-analysis was performed using random effects with the DerSimonian−Laird method for estimation of tau2; NAFLD, nonalcoholic fatty liver disease; CI, confidence interval; SE, standard error.

Twenty studies (14 in adults and 6 in children) were eligible to estimate the relationship between rs58542926 and NAFLD. Data from adults (15,901 individuals) found that the rs58542926 T allele was positively associated with NAFLD (OR=1.62; 95% CI: 1.40, 1.86; *P*<0.01, **Figure 2**). Children (3544 individuals) had a stronger association with NAFLD (OR=2.87; 95% CI: 1.85, 4.46; *P*<0.01). The effect value of children was statistically significantly higher than that of adults (*P*=0.01).

### The rs58542926 T allele increased the risk of steatosis and fibrosis in adults

Seven studies (3,461 adults) were eligible for estimation of the relationship between rs58542926 and steatosis. Steatosis progression showed a positive association with the rs58542926 T allele in 6 studies (MD=0.22; 95% CI: 0.05, 0.39; *P*=0.01; **Figure S2**). The presence of severe steatosis (stage S0-S1 versus stage S2-S3) showed a positive association with the rs58542926 T allele in 4 studies (OR=1.52; 95% CI: 1.14, 2.02; *P*<0.01; **Figure S3**).

**Figure 3:**
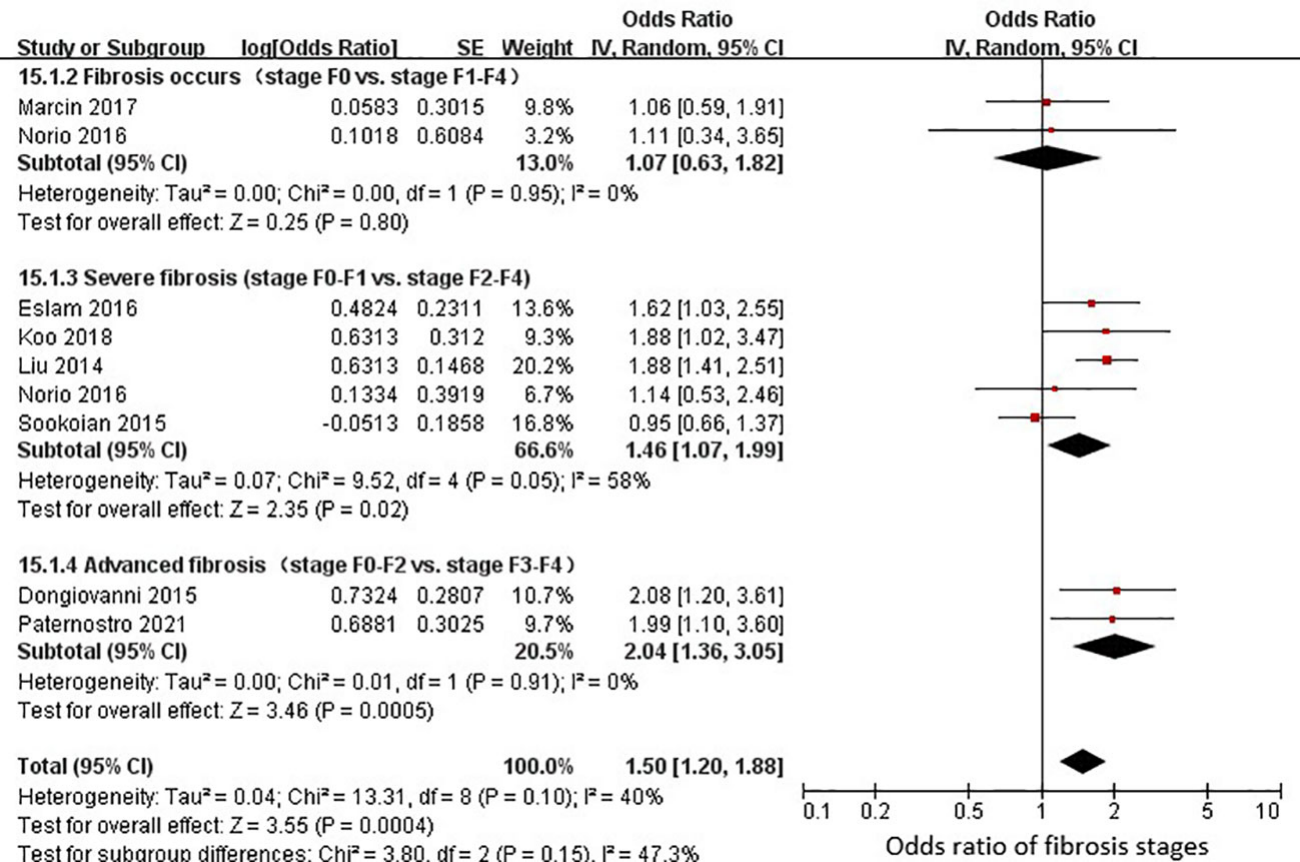
rs58542926 T allele was associated with a higher odds ratio with fibrosis stages. Data from 4928 individuals with liver biopsy. The rs58542926 T allele was positively associated with severe and advanced fibrosis stages (using a dominant model of inheritance). Meta-analysis was performed using random effects with the DerSimonian−Laird method for estimation of tau^2^; CI, confidence interval; SE, standard error.

Nine studies (4928 adults) were eligible for an estimation of the relationship between rs58542926 and fibrosis. A positive association between fibrosis progression and the rs58542926 T allele was detected in 4 studies (MD=0.32; 95% CI: 0.03, 0.61; *P*=0.03; **Figure S4**). Data from 8 studies found that the rs58542926 T allele was associated with fibrosis stages (OR=1.50; 95% CI: 1.20, 1.88; *P*<0.01; **Figure 3**). Given the disparities between different fibrosis stages, subgroup comparisons (with fibrosis vs without fibrosis; fibrosis stage 0-1 vs 2-4; fibrosis stage 0-2 vs 3-4) were also conducted. No significant difference was observed among fibrosis stages (OR=1.07; 95% CI: 0.63, 1.82; *P*=0.80; **Figure 3**). However, the rs58542926 T allele had positive associations with severe (OR=1.46; 95% CI: 1.07, 1.99; *P*=0.02; **Figure 3**) and advanced fibrosis (OR=2.04; 95% CI: 1.36, 3.05; *P*<0.01; **Figure 3**). In the sensitivity analysis conducted with omission of nonliver biopsy studies, consistent results were observed.

### The rs58542926 T allele was associated with increased ALT in both adults and children

Associations for plasma levels of ALT and AST were extracted from 19 studies (15 in adults and 4 in children) and 15 studies (13 in adults and 2 in children), respectively. ALT and AST were significantly associated with rs58542926 in adults (MD=3.34; 95% CI: 1.67, 5.00; *P*<0.01; **Figure S5**; MD=1.91; 95% CI: 0.59, 3.23; *P*<0.01; **Figure S6**). In children, a positive relationship was observed for the rs58542926 T allele and ALT (MD=3.93; 95% CI: 1.71, 6.16; *P*<0.01; **Figure S5**). No relationship was observed for the variant and AST in children (MD=2.90; 95% CI: -1.58, 7.37; *P*=0.20; **Figure S6**). The differences in effect sizes between children and adults were not statistically significant for ALT or AST (both *P*>0.05).

### The rs58542926 T allele was negatively associated with TC, TG, and LDL in both adults and children

**Figure 4:**
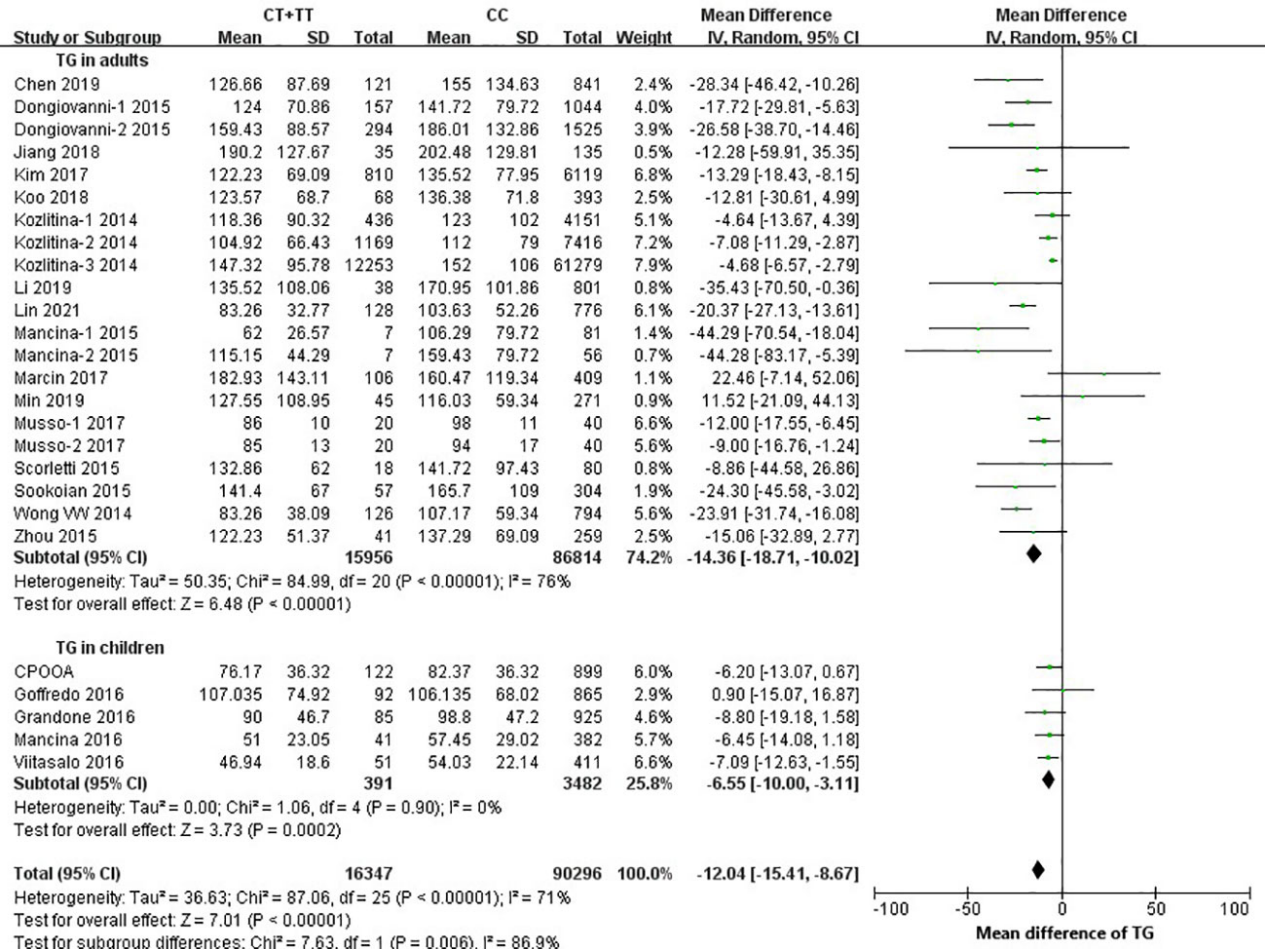
rs58542926 T allele associated with lower TG. Data from 106,643 individuals (102,770 adults, 3873 children). rs58542926 T allele was positively associated with TG in both adults and children (using a dominant model of inheritance), where data represent SD change in TG (mg/dl) per T-allele. Meta-analysis was performed using random effects with the DerSimonian−Laird method for estimation of tau^2^; TG, triglyceride; CI, confidence interval; SE, standard error.

Twenty-one studies (16 in adults and 5 in children) were eligible for estimation of the relationship between rs58542926 and serum lipid levels. Negative relationships were observed for the rs58542926 T allele and TC (MD=-10.01; 95% CI: -12.46, -7.56; *P*<0.01; MD=-10.85; 95% CI: -14.64, -7.05; *P*<0.01; **Figure S7**), TG (MD=-14.36; 95% CI: -18.71, -10.02; *P*<0.01; MD=-6.55; 95% CI: -10.00, -3.11, *P*<0.01; **Figure 4**), and LDL (MD=-2.04; 95% CI: -2.63, -1.44; *P*<0.01; MD=-8.77; 95% CI: -11.25, -6.30; *P*<0.01; **Figure S8**) in pooled analyses in both adults and children. Data from 19 studies (15 in adults and 4 in children) found that rs58542926 was not associated with HDL in adults and children (MD=0.66; 95% CI: 0.00, 1.32; *P*=0.05; MD=-0.81; 95% CI: -2.16, 0.53, *P*=0.24; **Figure S9**). In addition, the serum levels of LDL and HDL were much lower in children than in adults (both *P*<0.05), and the serum level of TG was much lower in adults (*P*<0.01), but the difference was not statistically significant for TC (*P*=0.72).

### Stability and publication biases of the present study

In the stability analysis of omitting one study each time and pooling the results of the remaining studies, the trend of associations was not strongly altered, indicating that the results of the pooled meta-analysis were stable. Potential publication bias was assessed by funnel plots in the present meta-analysis. The funnel plots were generally symmetrical, indicating that the results are unlikely to be severely affected by publication bias (**Figure S10**, **S11**).

## Discussion

### Main results

In this meta-analysis, we confirmed the *TM6SF2* rs58542926 T allele as a risk factor for the susceptibility and development of NAFLD and its related metabolic phenotypes in adults and children. Nonetheless, the rs58542926 T allele was a protective factor for serum lipid levels. In the present study, it is noteworthy that carrying the T allele is associated with a higher risk of NAFLD in children than in adults. Compared with adults, the rs58542926 T allele had a stronger effect size in pediatric NAFLD. Previous studies have shown that compared with adults, children with NAFLD may progress more rapidly and are more likely to develop fibrosis and end-stage liver disease in early adulthood (55). In addition, NAFLD will also increase the risk of multiple complications, such as hypertension, metabolic syndrome and diabetes (55, 56).

*TM6SF2* was first reported in 2000 (9), and it contains 10 transmembrane domains. It is mainly expressed in the small intestine and liver, which are associated with lipid metabolism. Kozlitina et al. (13) showed that TM6SF2 is an endoplasmic reticulum membrane protein and that the *TM6SF2* rs58542926 variant (C>T) encodes a glutamate to lysine at amino acid 167 (E167K). TM6SF2 is involved in the VLDL-TG secretion pathway (inactivation of *TM6SF2*, a gene defective in fatty liver disease, impairs lipidation but not secretion of VLDL) (57). The E167K variant leads to misfolding and increases the degradation of TM6SF2 in hepatocytes and induces an increase in liver TG content and a decrease in circulating lipids (13, 58).

Our results showed that rs58542926 was related to steatosis progression, severe steatosis, fibrosis stages and fibrosis progression in adults. However, there was no statistically significant difference in the fibrosis stages. Only two studies investigated the occurrence of fibrosis with a relatively small sample size, which was inadequate to reveal the real association. In previous studies, the relationship of *TM6SF2* rs58542926 with liver fibrosis has been controversial. The discrepancy between these results may be related to the different methods of hepatic steatosis and liver fibrosis examinations used in the studies, such as ultrasound (26, 34, 59), magnetic resonance imaging (25, 40), and liver biopsy (10, 22, 29), among others. Research has shown that the sensitivity of ultrasound decreases when the liver fat is less than 30% (60). In the present study, the meta-analyses of steatosis and fibrosis analyses only included studies which used the biopsy to avoid measurement bias.

AST and ALT are biomarkers of liver injury. ALT is mainly distributed in the cytoplasm of hepatocytes, and the increase in ALT reflects damage to the hepatocyte membrane and is the most sensitive biomarker (61). The present study only found that the adult T allele carriers had higher AST. NAFLD is a chronic progressive disease, and the degree of liver injury is relatively low in children. A significant increase in AST often occurs in patients with more severe hepatocyte destruction. Therefore, it seems reasonable that there was no relationship between AST and rs58542926 in children. Intriguingly, the genetically engineered transgenic mouse model showed that alternation of ALT or AST was not detected in the liver transgenic Alb-*TM6SF2* mice (overexpression of humanTM6SF2) or in the *tm6sf2* knockout mice (62). Therefore, the correlation between *TM6SF2* rs58542926 and ALT and AST in present study may be mainly attributed to the chronic liver injury caused by rs58542926 indirectly. At present, the gold standard for the diagnosis of NAFLD is liver biopsy, but it is expensive and invasive. The common screening method for pediatric NAFLD is ALT with or without liver ultrasonography (63–65). As an effective and reversible disease for early intervention, there are still some disputes about the sensitivity of screening children with NAFLD using ALT as a biomarker. However, studies have confirmed the reliability of using serum ALT to screen overweight children for NAFLD in primary health care (66). Our meta-analysis further confirmed the association between TM6SF2 rs58542926 and ALT. This is in line with the recommendations of the American Academy of Pediatrics on NAFLD screening for children over 10 years of age with overweight or obesity risk factors (67) and may provide a reference for the early detection of NAFLD in children.

Based on the molecular function of TM6SF2, it is logical to observe lower levels of TG, TC and LDL in serum among T allele carriers. TM6SF2 transfers TG from cytoplasmic to VLDL particles (57). Overexpression of *TM6SF2* decreased the number and size of lipid droplets. Overall, the trends of serum lipid levels were consistent in both adults and children. In addition, animal models showed that overexpression of human *TM6SF2* in mice could increase serum lipid levels, including TC, LDL and TG (62, 68). When *tm6sf2* was knocked down or knocked out in mice, the circulating lipid level decreased as well (62, 69). However, *TM6SF2* rs58542926 was not associated with HDL in the present study. The synthesis, assembly and transport of HDL particles are different from those of lipoproteins carrying LDL and TG (70). The associations between serum lipid levels are consistent with the results of Pirola et al., who also did not detect an association between HDL and rs58542926 (70). The variant *TM6SF2* rs58542926 could increase liver lipoprotein accumulation and reduce the concentration of serum lipids in peripheral serum plasma (71). Therefore, the *TM6SF2* rs58542926 T allele not only increases the risk of NAFLD but also has a protective effect against cardiovascular disease (70). Interestingly, in present study, the effect value differences between TG and LDL in adults and children showed the opposite trend: in TG, the effect value of adults was higher than that in children, while in LDL, the effect value in children was higher than that in adults. As the disease progresses in adulthood, the transport function of lipid droplets was probably further affected, so the TG serum levels in adults might have been substantially reduced in comparison with those in children. Endogenous TG in serum is mainly transported by VLDL. With the transfer of TG from VLDL, the change in molecular composition can turn VLDL into intermediate density lipoprotein (IDL). When the cholesterol content in IDL is higher than that in TG, IDL will become LDL. Therefore, the effect value of LDL in children carrying the T allele seems higher than that in adults. To be mentioned, children with NAFLD included in the present analyses were mainly children with overweight/obesity. This phenomenon alerts that even children with overweight/obesity and favorable serum lipid levels are still at risk for NAFLD. Ultrasonography and/or transient elastography should be implemented with overweight or obese children as recommended by the European Society for Pediatric Gastroenterology, Hepatology, and Nutrition (ESPGHAN) (65).

### Strengths and limitations.

Previous meta-analyses conducted between *TM6SF2* rs58542926 and NAFLD mainly focused on the sole association between rs58542926 and susceptibility to NAFLD or aminotransferase or serum lipid levels or the progression of NAFLD or some phenotypes (12, 70, 72–74). To our knowledge, the present study is the first meta-analysis that included both children and adults to reveal the effects of rs58542926 on the spectrum of NAFLD and its related metabolic phenotypes. Furthermore, we dissected the effect size between adults and children.

Nevertheless, some limitations need to be mentioned as well. First, the meta-analysis results were based on unadjusted pooling of previous findings due to the lack of raw data for eligible studies. Second, for consistency in the summary of results, in some studies, some values of medians and the interquartile range or range were converted to the mean and SD, which could cause error in the estimate. However, we used the improved estimation methods of Wan et al. (75) and Luo et al. (76) to reduce bias as much as possible. Third, there was no hierarchical adjustment for different ethnic groups, but the fixation index did not show disparities in rs58542926 among different ethnic groups. Therefore, the impact of ethnic differences would be weak. Last, the disparities between adults and children might be caused by the heterogeneities among different studies; however, assessing the credibility of an apparent effect modification is challenging (77).

## Conclusions

*TM6SF2* rs58542926 was associated with the incidence and progression of NAFLD and its related metabolic phenotype in both adults and children. The rs58542926 SNP had stronger associations with NAFLD, TG and LDL in children than in adults, which indicated that specific health education and liver ultrasound screening should start in children who are susceptible to NAFLD (e.g., children with obesity carrying rs58542926 CT or TT and with favorable serum lipid levels).

## Data availability statement

The original contributions presented in the study are included in the article/**Supplementary Material**. Further inquiries can be directed to the corresponding author.

## Author contributions

Study concept and design: HW, H-JW. Methodology: X-YL, ZL. Collection of data: X-YL, LL. analysis and interpretation of data: X-YL, ZL.Drafting of the manuscript: X-YL, HW. Comprehensive review and editing: HW. Critical revision of the manuscript for important intellectual content: all authors. Funding acquisition: HW. All authors have read and approved the published manuscript. All authors contributed to the article and approved the submitted version.

## Funding

The present research was funded by Peking University Medicine Fund of Fostering Young Scholars’ Scientific & Technological Innovation (BMU2022PY018), the Fundamental Research Funds for the Central Universities and the Sino-German Mobility Programme (M-0015).

## Acknowledgments

We thank Dr. Maike Wolters, Leibniz Institute for Prevention Research and Epidemiology - BIPS, for reviewing and commenting on the manuscript.

## Conflict of interest

The authors declare that the research was conducted in the absence of any commercial or financial relationships that could be construed as a potential conflict of interest.

## Publisher’s note

All claims expressed in this article are solely those of the authors and do not necessarily represent those of their affiliated organizations, or those of the publisher, the editors and the reviewers. Any product that may be evaluated in this article, or claim that may be made by its manufacturer, is not guaranteed or endorsed by the publisher.
