# Supplementary material for: TM6SF2 rs58542926 is related to hepatic steatosis, fibrosis and serum lipids both in adults and children: A meta-analysis
Source: Front Endocrinol (Lausanne). 2022 Oct 24;13:1026901. doi: 10.3389/fendo.2022.1026901 (PMC9637980; doi:10.3389/fendo.2022.1026901)
Supplement: Supplementary file 1 [file DataSheet_1.docx]

Supplementary Material

**Table S1 The characteristics of the included studies in this meta-analysis**

| First author, year | Country/Region | Population type | Sample size | Genotypes (CC/CT+TT) | NAFLD  Detection methods |
| --- | --- | --- | --- | --- | --- |
| Arslanow,2016 | Germany | Adult | 348 | 139/35 | TE |
| Bale,2017 | India | Adult | 503 | 318/112 | Ultrasound |
| Chen,2019 | China | Adult | 963 | 841/121 | Ultrasound |
| Danford,2018 | America | Adult | 177 | 140/36 | Biopsy |
| Di Costanzo,2018 | Caucasus | Adult | 413 | 375/38 | Ultrasound |
| Dongiovanni,2015 | Europe/Sweden | Adult | 3020 | 2569/451 | Biopsy |
| Eslam,2016 | Caucasus | Adult | 502 | 391/111 | Biopsy |
| Jiang,2018 | America | Adult | 170 | 135,35 | Biopsy |
| Kalafati,2019 | Greece | Adult | 351 | 331/20 | Ultrasound |
| Kawaguchi,2018 | Japan | Adult | 8573 | 7279/1294 | Biopsy |
| Krawczyk,2016 | Caucasus | Adult | 143 | 110/33 | Ultrasound |
| Kim,2017 | Finland | Adult | 6929 | 6119/810 | - |
| Koo,2018 | South Korea | Adult | 461 | 393/68 | Ultrasound, Biopsy |
| Li,2019 | China | Adult | 839 | 801/38 | Ultrasound |
| Lin,2021 | Hong Kong, China | Adult | 904 | 776/128 | MRI-PDFF, TE |
| Lisboa,2020 | Brazil | Adult | 285 | 148/137 | Ultrasound, Biopsy |
| Liu,2014 | Caucasus | Adult | 1074 | 945/129 | Biopsy |
| Liu Y,2014 | Caucasus | Adult | 728 | 599/129 | Biopsy/Ultrasound |
| Marcin,2017 | Germany | Adult | 515 | 409/106 | Biopsy |
| Mancina,2015 | Europe | Adult | 151 | 137/14 | Biopsy/MRS |
| Min,2019 | China | Adult | 316 | 271/45 | FibroScan |
| Musso,2017 | Italy | Adult | 120 | 80/40 | Biopsy |
| Norio,2016 | Japan | Adult | 139 | 104/35 | Biopsy |
| Paternostro,2021 | Europe | Adult | 703 | 588/115 | Biopsy |
| Petta,2018 | Italy | Adult | 890 | 820/70 | FibroScan |
| Scorletti,2015 | America | Adult | 98 | 80/18 | Ultrasound/CT/MRI |
| Sookoian,2015 | Argentina | Adult | 361 | 304/57 | Biopsy |
| Wang,2016 | China | Adult | 768 | 635/98 | Ultrasound |
| Zhou,2015 | Finland | Adult | 300 | 259/41 | MRS/Biopsy |
| Wong VW,2014 | Hong Kong, China | Adult | 920 | 794/126 | MRS, TE |
| Kozlitina J,2014 | America/Denmark | Adult | 86704 | 72846/1870 | - |
| Goffredo,2016 | Caucasus | Child | 957 | 865/92 | MRI/Biopsy |
| Grandone,2016 | Italy | Child | 1000 | 925/85 | Ultrasound |
| Lin,2016 | Taiwan, China | Child | 825 | 719/106 | Ultrasound |
| Mancina,2016 | Italy | Child | 423 | 382/41 | Ultrasound |
| Viitasalo,2016 | Finland | Child | 462 | 411/51 | - |
| Zusi,2019 | Italy | Child | 514 | 493/21 | Ultrasound |
| Di Costanzo,2019 | Caucasus | Child | 230 | 198/32 | MRI |
| CPOOA | China | Child | 1027 | 899/122 | Ultrasound |

TE, transient elastography; MRI-PDFF, magnetic resonance imaging-derived proton density fat fraction; MRS, proton-magnetic resonance spectroscopy; MRI, Magnetic Resonance Imaging


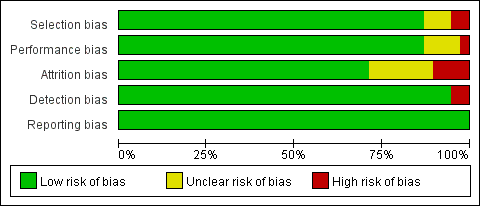


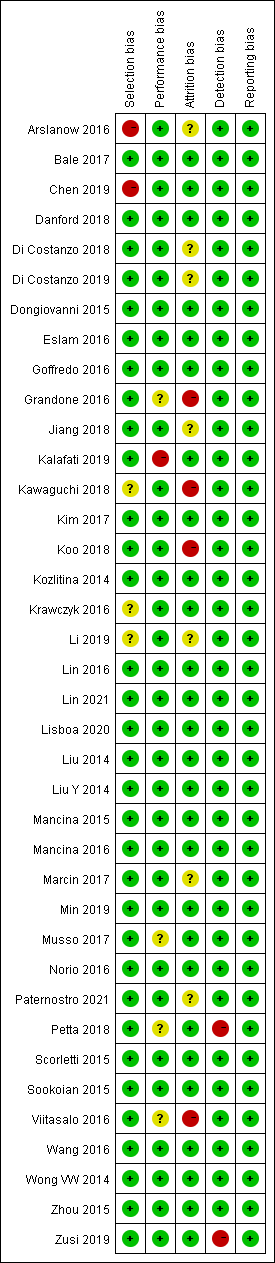


**Figure S1 Risk of bias summary for this meta-analysis**


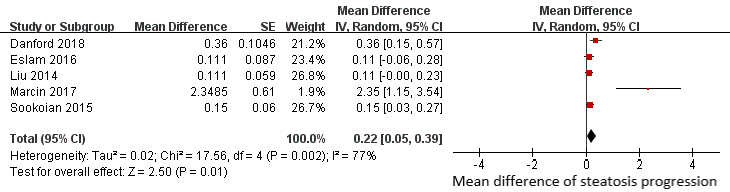


**Figure S2 Association between rs58542926 C>T and steatosis progression.** Data from 2619 adults with liver biopsy. rs58542926 C>T was positively associated with steatosis stage (using a dominant model of inheritance). Meta-analysis was performed using random effects with the DerSimonian-Laird method for estimation of tau2; CI, confidence interval; SE, standard error.


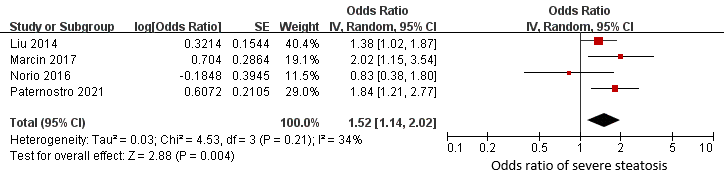


**Figure S3 Association between rs58542926 C>T and the presence of severe steatosis (stage S0-S1 versus stage S2-S3)**. Data from 2431 adults with liver biopsy. rs58542926 C>T was positively associated with the presence of severe steatosis (using a dominant model of inheritance). Meta-analysis was performed using random effects with the DerSimonian-Laird method for estimation of tau^2^; CI, confidence interval; SE, standard error.


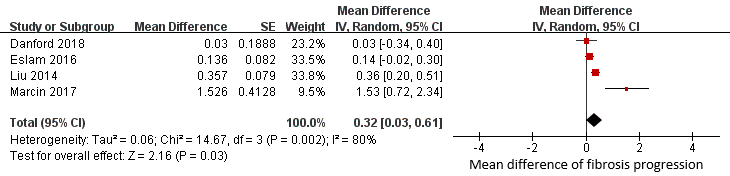


**Figure S4 Association between rs58542926 C>T and fibrosis progression**. Data from 2073 adults with liver biopsy. rs58542926 C>T was positively associated with fibrosis stage (using a dominant model of inheritance). Meta-analysis was performed using random effects with the DerSimonian-Laird method for estimation of tau^2^; CI, confidence interval; SE, standard error.


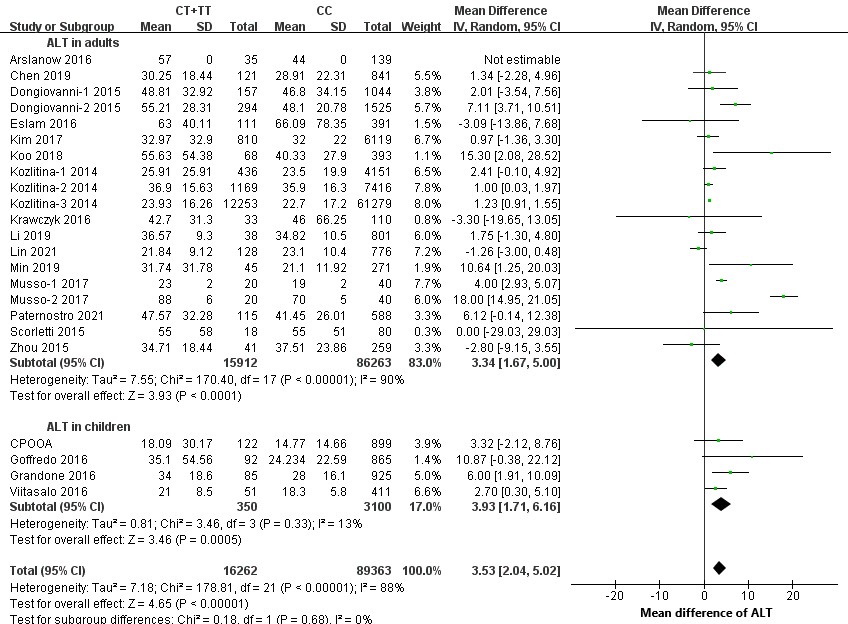


**Figure S5: rs58542926 T allele** **associated with higher ALT.** Data from 105,625 individuals (102,175 adults, 3450 children). rs58542926 T allele was positively associated with ALT both in adults and children (using a dominant model of inheritance), where data represent SD change in ALT (IU/L) per T-allele. Meta-analysis was performed using random effects with the DerSimonian‒Laird method for estimation of tau2; ALT, alanine aminotransferase; CI, confidence interval; SE, standard error.


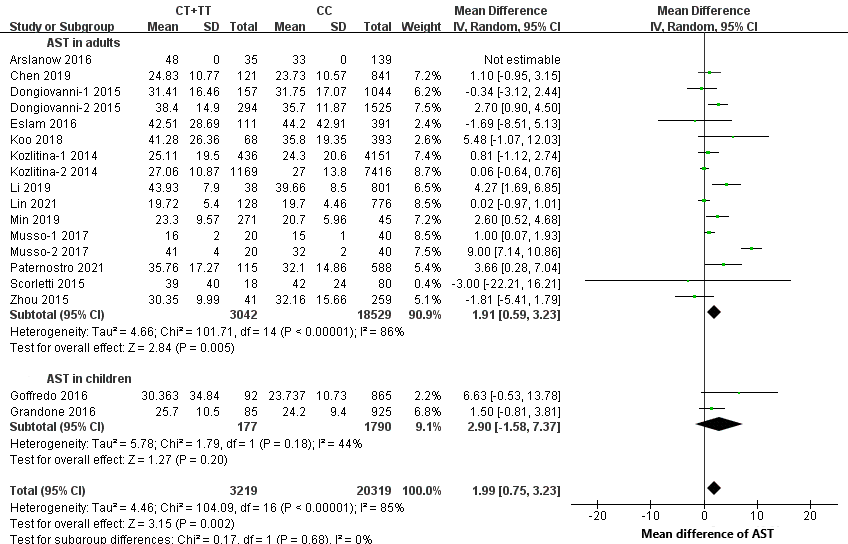


**Figure S6 Association between rs58542926 C>T and AST.** Data from 23538 individuals (21571 adults, 1967 children). Rs58542926 C>T was positively associated with AST in adults (using a dominant model of inheritance), where data represent SD change in AST (IU/L) per T-allele. Meta-analysis was performed using random effects with the DerSimonian-Laird method for estimation of tau2; AST, aspartate aminotransferase; CI, confidence interval; SE, standard error.


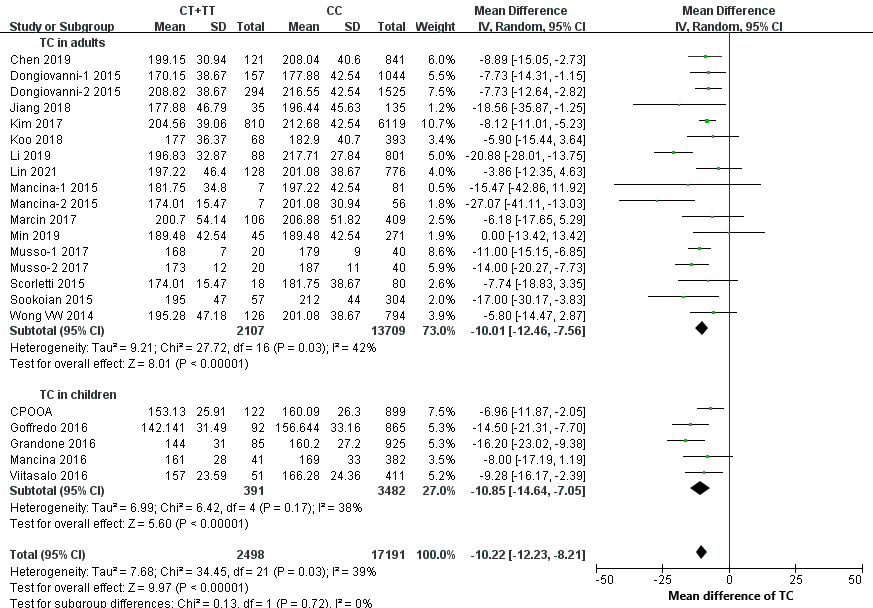


**Figure S7 Association between rs58542926 C>T and TC.** Data from 19689 individuals (15816 adults, 3873 children). rs58542926 C>T was positively associated with TC both in adults and children (using a dominant model of inheritance), where data represent SD change in TC (mg/dl) per T-allele. Meta-analysis was performed using random effects with the DerSimonian-Laird method for estimation of tau^2^; TC, total cholesterol; CI, confidence interval; SE, standard error.


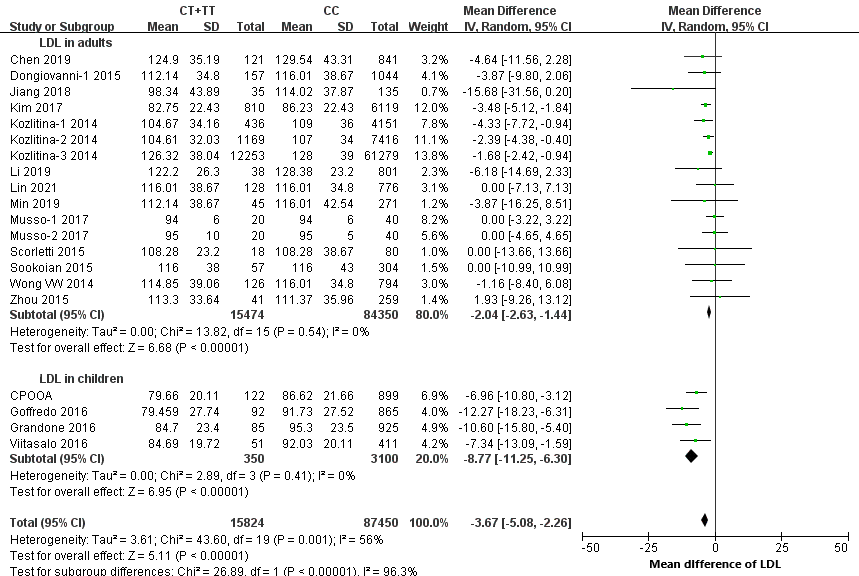


**Figure S8 Association between rs58542926 C>T and LDL.** Data from 103274 individuals (99824 adults, 3450 children). rs58542926 C>T was positively associated with LDL both in adults and children (using a dominant model of inheritance), where data represent SD change in LDL (mg/dl) per T-allele. Meta-analysis was performed using random effects with the DerSimonian-Laird method for estimation of tau^2^; LDL, low-density-lipoprotein; CI, confidence interval; SE, standard error.


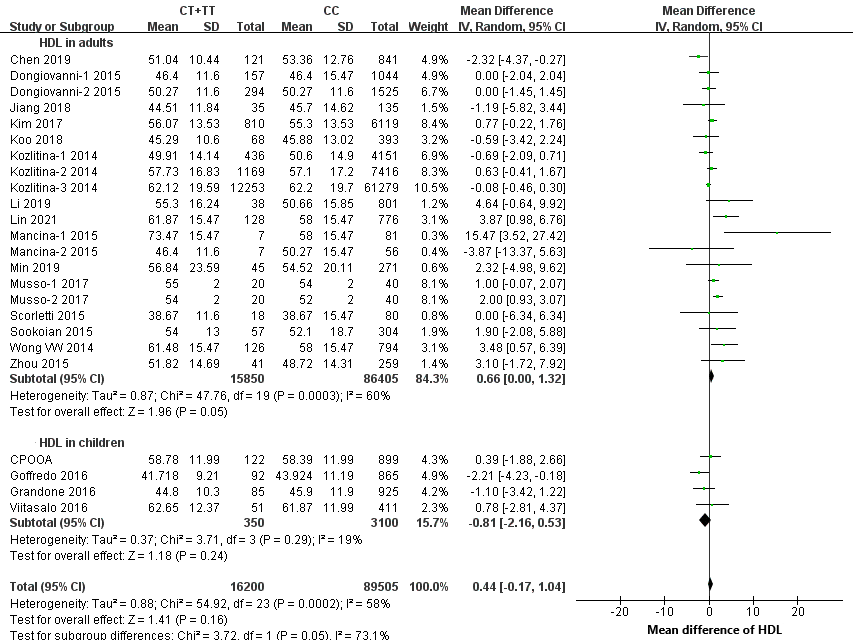


**Figure S9 Association between rs58542926 C>T and HDL.** Data from 105705 individuals (102255 adults, 3450 children). rs58542926 C>T was positively associated with HDL both in adults and children (using a dominant model of inheritance), where data represent SD change in HDL (mg/dl) per T-allele. Meta-analysis was performed using random effects with the DerSimonian-Laird method for estimation of tau^2^; HDL, high-density-lipoprotein; CI, confidence interval; SE, standard error.


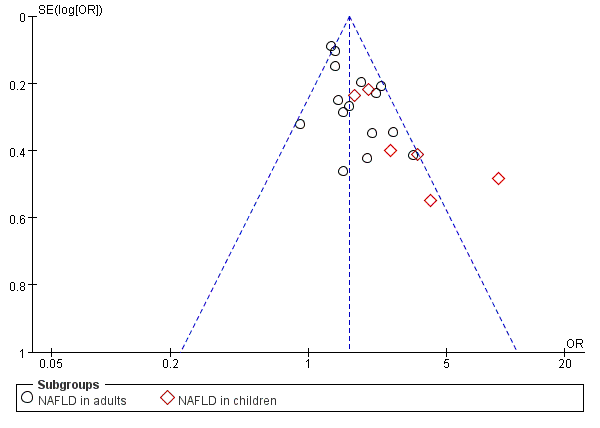


**Figure S10 The funnel plot of NAFLD research with pseudo 95% confidence limits**


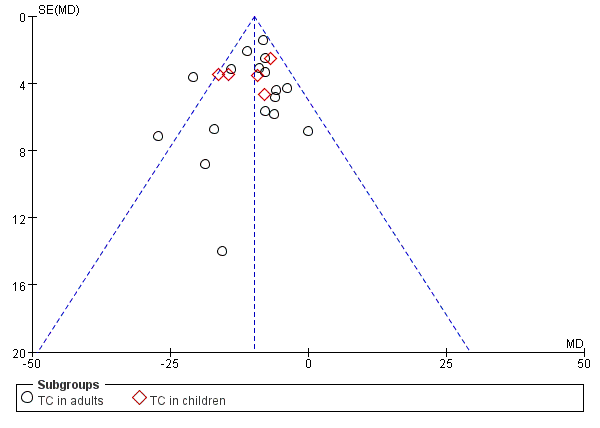


**Figure S11 The funnel plot of TC research with pseudo 95% confidence limits**
